# The Comparative Outcomes of Particulate Versus Coil Preoperative Embolization for Carotid Body Tumors

**DOI:** 10.3389/fonc.2022.860788

**Published:** 2022-05-25

**Authors:** Xun Huang, Lin Wang, Yangjing Chen, Jianlin Liu, Yamin Liu, Lin Yang

**Affiliations:** ^1^Department of Vascular Surgery, The First Affiliated Hospital of Xi’an Jiaotong University, Xi’an, China; ^2^Department of Critical Care Medicine, Xi’an Traditional Chinese Medicine Hospital, Xi’an, China; ^3^Department of Otolaryngology, The First Affiliated Hospital of Xi’an Jiaotong University, Xi’an, China; ^4^Department of Interventional Radiology, The First Affiliated Hospital of Xi’an Jiaotong University, Xi’an, China

**Keywords:** particulate and coil embolization for CBTs carotid body tumor, embolization, outcomes, particulate, coil

## Abstract

Till now, the effect of different embolic materials (particulate vs coil) on pre-embolization of carotid body tumors remains poorly understood. The aim of this study was to explore the comparative results between particulate and coil embolization for carotid body tumors. Thirty-seven patients with carotid body tumors who underwent embolization before surgical resection were reviewed and analyzed in this retrospective study between 2008 and 2020. Twenty-one patients were included in the particulate group, while 16 patients were included in the coil group. All procedure-related details, complications and 5-year follow-up data were collected in the study. The preoperative embolization time was obviously longer in the particulate group than in the coil group (42.6 ± 12.3 min vs. 33.7 ± 10.1 min, *P* =.02), and the fluo time of the procedure (864.5 ± 240.9 s vs. 729.6 ± 251.5 s) and cumulative air kerma (634.6 ± 188.4 mGy vs. 486.7 ± 164.7 mGy) value were higher in the particulate group (*P* =.01). The incidences of total adverse events in both groups were not significantly different (28.6% vs. 25.0%, *P* >.05); however, two cases of ectopic embolization only occurred in the particulate group. Interestingly, medical expenditure was higher in the particulate group than in the coil group (*P* =.02). For the 3-year follow-up evaluation, recurrence and all-cause mortality were similar in both groups (*P* >.05). Preoperative embolization with coils could be relatively safe, have a lower radiation dosage and be cost-effective for the treatment of carotid body tumors.

## Introduction

Carotid body tumors (CBTs) are relatively rare neoplasms (incidence rate one in 30 000) that usually grow slowly, and most are benign (1). Surgical resection of CBTs is currently the gold standard for the treatment of disease, but the operation can frequently be tricky because of the adjacent neurovascular structures that may be involved in the tumors, especially in the case of advanced Shamblin classification (1, 2). Excessive blood loss and cranial nerve damage may be the most worrying complications during the operation; excessive intraoperative bleeding may increase the chances of cranial nerve injury and thus complicate the operation (3, 4). Preoperative embolization prior to surgical excision of CBTs has been reported to reduce blood loss and thus provide better operational visualization, facilitate tumor excision and decrease morbidity related to perioperative complications (5, 6). However, the embolization of the feeding arteries of CBTs can also cause some unwanted complications, such as ectopic embolization or stroke (3, 4).

Preembolization of CBTs can be carried out by delivering agents such as particulates or coils into the feeding arteries of the tumors *via* a microcatheter. The preoperative embolization technique has been proven to be effective in the devascularization of CBTs before surgical resection in previous studies (7–10). Several materials have been successfully applied in the preoperative embolization of CBTs, such as gelatin microspheres, nonspherical polyvinyl alcohol particles, coils, Onyx and gelfoam (8–10). However, discrepancies in the safety and efficacy of these materials have rarely been reported in the literature. The aim of this study was to retrospectively compare different embolization agents, such as particulates or coils, in the preoperative embolization of CBTs at our institution.

## Methods

### Baseline Data

**Figure 1:**
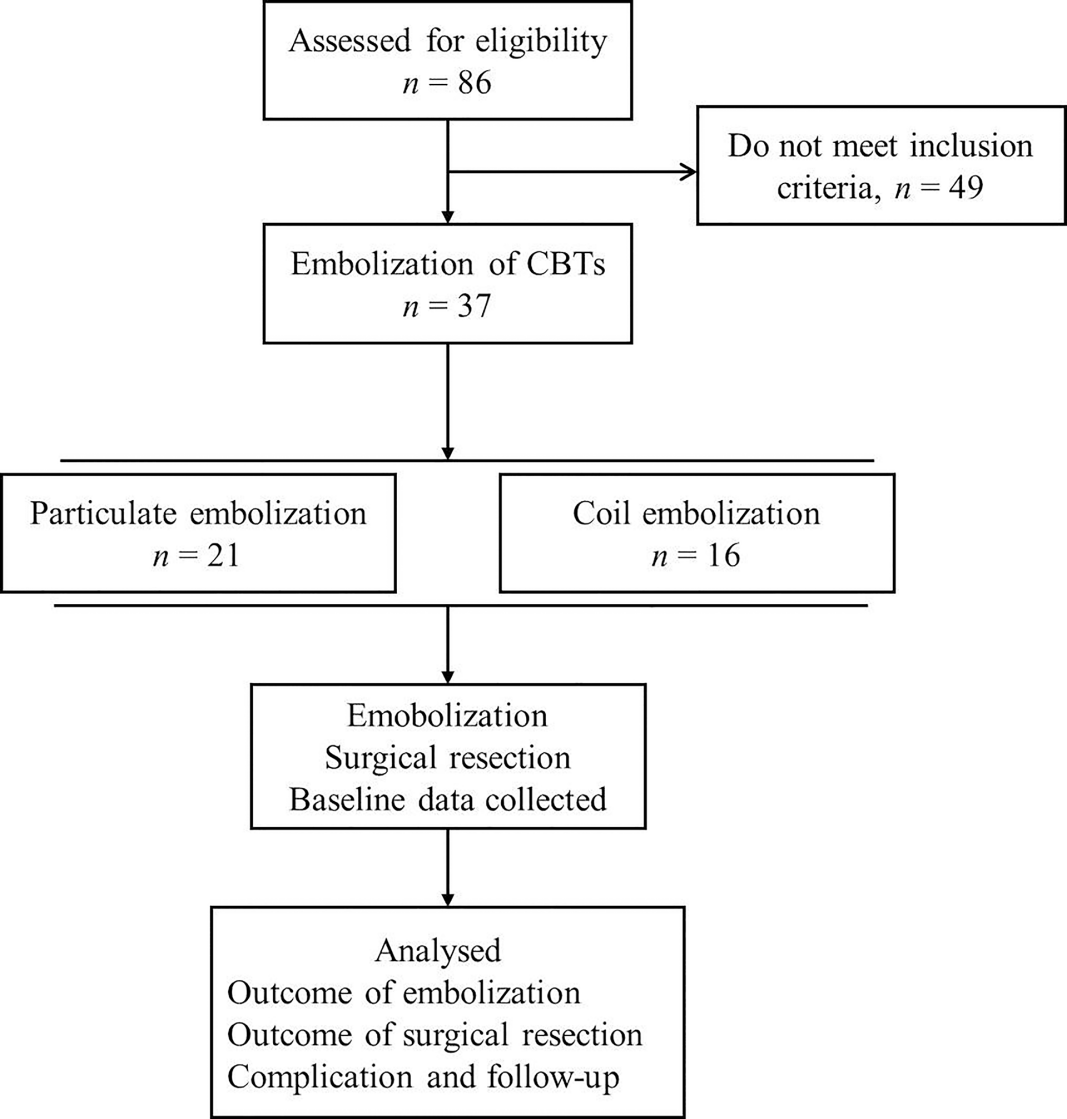
The flowchart of this study. CBTs, Carotid body tumors.

Cases of preoperative embolization of CBTs were retrospectively reviewed at our institution from 2008 to 2020 (**Figure 1**). The clinical data of CBTs classified as Shamblin type II and III were included in the study, while tumors of type I were excluded (11). The clinical manifestations and vascular images of patients were reviewed by a radiologist and a vascular surgeon, respectively, with at least 10 years of clinical experience. Thirty-seven patients were divided into two groups based on the embolization materials used during the procedure. Twenty-one patients were included in the particulate group, while 16 patients were included in the coil group. Patients who had both particulate and coil devascularization or had bilateral tumors were excluded. This retrospective study was approved by the ethics committee and review board of the First Affiliated Hospital of Xi’an Jiaotong University. Written informed consent was obtained from all patients.

### Pre-Embolization Procedure

The preoperative embolization procedures were performed under local anesthesia 1 or 2 days before the surgical resection of CBTs. A 5-French guide catheter was placed into the common carotid artery *via* femoral artery access, and digital subtraction angiography (DSA) of the external and internal carotid arteries was performed. Then, a microcatheter was advanced through the guide catheter under the roadmap into the main trunk of the tumor-feeding artery. Devascularization of the tumor was then performed with polyvinyl alcohol or gelatin microsphere particles ranging from 100~500 μm or various coils (Cook, Indiana, USA) through the microcatheter until stasis of the feeding artery. DSA was performed to confirm the devascularization effect of the tumor after the embolization procedure.

### Surgical Excision Procedure

After the embolization procedure, surgical resection of the tumor was carried out as scheduled. The procedure was performed under general anesthesia. A longitudinal incision along the sternomastoid muscle was made for exposure of the tumor. The common facial vein was normally cut off and ligated. Then, the common carotid artery was identified and controlled. The carotid vessels were meticulously dissected from the entangling tumor. Sometimes parts of the adventitia of carotid arteries had to be removed for resection of the tumor. In the scenarios of carotid artery resection and reconstruction, autologous saphenous vein grafts or bovine pericardial patches were used.

### Evaluation and Follow-Up

Procedure details were specified with embolization time, radiation dosage, numbers of ectopic embolizations, volume of operative blood loss, resection time of the tumor, days of hospital stay and medical expenditure. Postoperative complications were evaluated, which included hematoma, nerve injury, incision dehiscence, fat liquefaction, incision infection. Stroke and death after procedure were defined as the major adverse events. Embolization and surgical operations were performed and evaluated by several skilled surgeons on our team with more than 10 years of experience in CBT operations. The evaluation indicators of angiography radiation safety were as follows: fluo time of procedure (FTP) and cumulative air kerma (CAK), which were confirmed based on the record of the angiography system.

Follow-up evaluation was carried out by our experienced team members with each patient followed for at least 3 years. CBT recurrence and all-cause mortality data were collected.

### Statistical Analysis

SPSS version 11.0 (SPSS, Chicago, IL) was used to analyze the data. Differences between groups were tested with the chi-square test or Fisher’s exact test to analyze the categorical data, and continuous variables were analyzed with Student’s *t* tests or analysis of variance (ANOVA) when the data were normally distributed. The Mann–Whitney *U* test was used when the data were not normally distributed. The reported *P* values were all 2-sided, and statistical significance was defined when *P* values were less than .05.

## Results

### The Characteristics of the Data Between Particulate and Coil Groups

Patient characteristics and baseline data are summarized in **Table 1**. The baseline data were not significantly different between the two groups, including sex, mean age, Shamblin classification and presenting symptoms. In addition, Shamblin type II tumors were relatively more common than type III tumors in all cases.

**Table 1:** The baseline data of the study.

	Particulate group (n = 21)	Coil group (n = 16)	*P* value*
Sex (female, %)	18 (85.7)	14 (87.5)	.63
Age (years)	54.3 ± 10.8	51.2 ± 9.3	.32^#^
Weight (kg)	62.4 ± 5.5	59.9 ± 5.1	.14^#^
Shamblin classification (%)			.62
II	13 (61.9)	10 (62.5)	
III	8 (38.1)	6 (37.5)	
Presenting symptoms (%)			
Mass	18 (85.7)	14 (87.5)	.63
Pain	4 (19.0)	3 (18.8)	.66
Dysphagia	2 (9.5)	1 (6.3)	.60
Dysphonia	1 (4.8)	1 (6.3)	.69
Fainting	2 (9.5)	1 (6.3)	.60
Horner’s syndrome	1 (4.8)	0 (0)	.57
Transient ischemic attack	2 (9.5)	1 (6.3)	.60

P value, compared with particulate group, * analysis via Fisher’s exact test; ^#^analysis via t test.

### Details of the Embolization Procedure in the Particulate and Coil Groups

Procedure details in the particulate and coil groups are shown in **Table 2**. The preoperative embolization time was obviously longer in the particulate group than that in the coil group (42.6 ± 12.3 min vs. 33.7 ± 10.1 min, *P* = .02), as well as the higher dosage of radiation during embolization procedure. The FTP (864.5 ± 240.9 s vs. 729.6 ± 251.5 s) and CAK (634.6 ± 188.4 mGy vs. 486.7 ± 164.7 mGy) values were higher in in the particulate group (*P* = .01). The adverse events of embolization were similar in both groups (**Table 2**); however, the two cases of ectopic embolization only occurred in the particulate group.

**Table 2:** Procedure details in particulate and coil groups.

	Particulate group (n = 21)	Coil group (n = 16)	*P* value*
Embolization time (min)	42.6 ± 12.3	33.7 ± 10.1	.02^#^
FTP (s)	864.5 ± 240.9	729.6 ± 251.5	.01^#^
CAK (mGy)	634.6 ± 188.4	486.7 ± 164.7	.01^#^
Ectopic embolization (%)	2 (9.6)	0 (0)	.32
Operative blood loss (ml)	132.6 ± 58.4	152.3 ± 53.6	.30^#^
Resection time of CBTs (min)	115.7 ± 34.9	102.6 ± 38.8	.29^#^
Hospital stay (d)	9.5 ± 3.6	10.1 ± 4.2	.64^#^
Medical expenditure (US dollars)	8158.9 ± 1763.6	6767.6 ± 1562.2	.02^#^

FTP, fluo time of procedure; CAK, cumulative air kerma; mGY, milligray; CBTs, carotid body tumors. P value, compared with PG group, *analysis via Fisher’s exact test; ^#^analysis via t test.

### Complication and Follow-Up Outcomes in the Particulate and Coil Groups

All patients underwent surgical resection after embolization (100%), and there were no significant differences in operative blood loss, resection time of tumors or hospital stay in either group. Complications after surgical resection and follow-up evaluation are listed in **Table 3**. There were no significant differences in hematoma (femoral artery puncture site), cranial nerve injury (temporary injury) and incision-related complications, all these complication recovered completely without further treatment. The incidences of stroke (major adverse event) in both groups were not significantly different (9.5% vs. 0%, *P* >.05), however, the stroke only occurred in the particulate group, one patient recovered completely after medicine therapy, and one patient remained with mild limb dysfunction. Interestingly, medical expenditure was higher in the particulate group than in the coil group (*P* = .02). Benign pathological findings accounted for 20 (95.2%) and 15 (93.8%) of the cases between the particulate and coil groups (*P *>.05)

**Table 3:** Complication and long-term outcomes in both groups.

	Particulate group (n = 21)	Coil group (n = 16)	*P* value*
Hematoma (%)	0 (0)	1 (6.3)	.43
Carotid repair (%)	2 (9.5)	0(0)	.32
Temporary nerve injury (%)	1 (4.8)	2 (12.5)	.40
Superior laryngeal nerve	1 (4.8)	1 (6.3)	
Recurrent laryngeal nerve	0 (0)	1 (6.3)	
Incision complications (%)	1 (4.8)	1 (6.3)	.69
Incision dehiscence	0 (0)	0 (0)	
Fat liquefaction	1 (4.8)	0 (0)	
Incision infection	0 (0)	1 (6.3)	
Major adverse events (%)	2 (9.5)	0 (0)	.32
Stroke (%)	2 (9.5)	0 (0)	.32
Death (%)	0 (0)	0 (0)	Null
Pathological benign (%)	20 (95.2)	15 (93.8)	.69
Recurrence (%)	0 (0)	0 (0)	Null
All-cause mortality (%)	1 (4.8)	0 (0)	.57

P value, compared with particulate group, *analysis via Fisher’s exact test.

No deaths or other severe complications occurred after the procedure. For the 3-year follow-up evaluation, no patient developed recurrence in both group, and one patient (4.8%) died due to cardiovascular diseases in the particulate group, but there were no significant differences in all-cause mortality in either group.

## Discussion

Carotid body tumors are rare tumors that belong to the paraganglioma system, the incidence is approximately 1:30 000 and accounts for 65% of paraganglioma of the head and neck (1, 11). Although most CBTs are benign, the possible malignant potential (the metastasis rate is about 5%) necessitates early and thorough surgical resection. However, a large amount of blood loss and other severe complications may occur in surgical excision, especially in Shamblin type II and III CBTs (12–14). Preoperative embolization of the tumor-feeding artery has been reported to aid in the successful surgical removal of tumors. Operative blood loss, resection time of the tumor, and hospitalization time can be decreased by preoperative angiography and tumor embolization (14–17). However, some other researchers found that preoperative embolization could not improve the outcomes of surgical resection of CBTs (15–17). In a previous study, the author found that preoperative embolization was beneficial to the surgical outcomes of CBTs, and no increased stroke rates or postoperative complications were observed (7). In this study, we confirmed that pre-embolization was a useful adjunct procedure for the surgical resection of CBTs, and the particulate and coil embolization material displayed a similar effect on the devascularization of CBT tumors.

Few studies, however, have been performed to explore the differences of those embolization agents in the surgical operations of CBTs. Multiple materials can be used for preoperative embolization, such as gelatin microspheres, nonspherical polyvinyl alcohol particles, coils, Onyx and gelfoam (10, 18–21). Some researchers even implanted covered stents into the external or internal carotid artery for preoperative embolization and proved the efficacy (8, 9). Although these materials can be adequate for devascularization of CBTs, complete embolization of the feeding artery is usually difficult to achieve. In addition, operative complications may occur during the devascularization of CBTs (2, 19), and our results were consistent with these studies.

In our study, we used polyvinyl alcohol (or gelatin microsphere) particles and coils for preoperative embolization of CBTs. We found that operative blood loss in the particulate group was not significantly different from that in the coil group. We presumed that particulates might disperse through the vasculature of CBT while coils could only block the main trunk of the feeding artery, but actually the embolization efficacy of those materials turned out to be almost the same. Furthermore, in a recent research without embolization (22), the mean intraoperative blood loss (750-800 ml) and operative time (140-150 min) were significantly high than our results (**Table 2**). The particulates were theoretically more liable to introduce ectopic embolization when compared to coils. Although no statistically significant differences were observed in these 2 groups, the cases suffering ectopic embolization only occurred in the particulate group. These data might indicate that coil embolization shows a better safety trend; however, this still needs further verification. However, what we need to emphasize is that there is no difference in the total effectiveness and adverse events index between the two technologies. Moreover, embolization combined with surgical resection does not increase the incidence of postoperative complications and stroke, and which was an adjunctive procedure to beneficial for CBT surgical outcomes (14); however, we also need to acknowledge that the surgical resection without embolization has achieved good surgical outcomes in experienced centers (22).

Furthermore, radiation damage and protection are some of the main concerns during endovascular therapy; however, there are no studies about the radiation dosage and potential injury of the pre-embolization of CBTs. In the present study, we found that the pre-embolization time was obviously longer in the particulate group than in the coil group, as well as the higher dosage of radiation (the fluo time and cumulative air kerma) during embolization in the particulate group. These data indicate that coil embolization may have a faster operation and better radiation safety, which has potential benefits for both patients and doctors. Normally, we have to spend extra time preparing particulate suspensions while the coils just save trouble, and the coil embolization operation is simpler; thus, it may save procedure time and reduce the radiation dosage. Moreover, medical expenditure was higher in the particulate group than in the coil group, and thus, the choice of coils for preoperative embolization might alleviate the financial burden of CBT patients.

### Limitation

Because the number of cases was limited in our study and a large randomized controlled trial might be difficult to conduct due to the rarity of CBTs, the comparative outcome of different embolization agents still needs to be confirmed in the future, and more data might be needed to validate our conclusion.

## Conclusions

In conclusion, our study concludes that preoperative embolization with coils could be relatively safe, have a lower radiation dosage and be cost-effective for the surgical treatment of CBTs.

## Data Availability Statement

The original contributions presented in the study are included in the article/supplementary material. Further inquiries can be directed to the corresponding author.

## Ethics Statement

The studies involving human participants were reviewed and approved by the First Affiliated Hospital of Xi’an Jiaotong University Institutional Review Board. The patients/participants provided their written informed consent to participate in this study.

## Author Contributions

Study design, XH, LW, YC, and LY. Data collection and data analysis, XH, LW, YC, JL, YL, and LY. Writing, XH, LW, YC, and LY. Revision and review manuscript, XH, LW, YC, JL, YL, and LY. All authors contributed to the article and approved the submitted version.

## Funding

This work was supported by the Natural Science Foundation of China (NO.8197 0365).

## Conflict of Interest

The authors declare that the research was conducted in the absence of any commercial or financial relationships that could be construed as a potential conflict of interest.

## Publisher’s Note

All claims expressed in this article are solely those of the authors and do not necessarily represent those of their affiliated organizations, or those of the publisher, the editors and the reviewers. Any product that may be evaluated in this article, or claim that may be made by its manufacturer, is not guaranteed or endorsed by the publisher.
